# *aP2*-Cre Mediated Ablation of GHS-R Attenuates Adiposity and Improves Insulin Sensitivity during Aging

**DOI:** 10.3390/ijms19103002

**Published:** 2018-10-01

**Authors:** Ligen Lin, Jong Han Lee, Ruitao Wang, Ru Wang, David Sheikh‐Hamad, Qun S. Zang, Yuxiang Sun

**Affiliations:** 1 Children’s Nutrition Research Center, Department of Pediatrics, Baylor College of Medicine, Houston, TX 77030, USA; ligenl@umac.mo (L.L.); jhleecw@gmail.com (J.H.L.); 2 State Key Laboratory of Quality Research in Chinese Medicine, Institute of Chinese Medical Sciences, University of Macau, Macau 999078, China; 3 College of Pharmacy, Gachon University, Incheon 21936, Korea; 4 Institute of Critical Care Medicine, Heilongjiang Academy of Medical Science, Heilongjiang 150081, China; ruitaowang@126.com; 5 Key Laboratory of Exercise and Health Sciences of Ministry of Education, Shanghai University of Sport, Shanghai 200438, China; wangru0612@163.com; 6 Department of Medicine, Baylor College of Medicine, Houston, TX 77030, USA; sheikh@bcm.edu; 7 Department of Surgery, University of Texas Southwestern Medical Center, Dallas, TX 75390, USA; qun.zang@utsouthwestern.edu; 8 Department of Nutrition and Food Science, Texas A&M University, College Station, TX 77843, USA

**Keywords:** ghrelin, GHS-R, tissue-specific knockdown mice, thermogenesis, UCP1, adipose tissues

## Abstract

Ghrelin via its receptor, the growth hormone secretagogue receptor (GHS-R), increases food intake and adiposity. The tissue-specific functions of GHS-R in peripheral tissues are mostly unknown. We previously reported that while GHS-R expression is very low in white and brown fat of young mice, expression increases during aging. To investigate whether GHS-R has cell-autonomous effects in adipose tissues, we generated *aP2*-Cre-mediated GHS-R knockdown mice (*aP2*-Cre/*Ghsr^f/f^*). We studied young (5–6 months) and old (15–17 months) *aP2*-Cre/*Ghsr^f/f^* mice and their age-matched controls. Interestingly, young *aP2*-Cre/*Ghsr^f/f^* mice had normal body weight but reduced fat; old mice showed pronounced reductions of both body weight and body fat. Calorimetry analysis revealed that *aP2*-Cre/*Ghsr^f/f^* mice had normal food intake and locomotor activity at both young and old age; but intriguingly, while energy expenditure was normal at young age, it was significantly increased at old age. Both young and old *aP2*-Cre/*Ghsr^f/f^* mice exhibited improved insulin sensitivity and glucose tolerance. Importantly, old *aP2*-Cre/*Ghsr^f/f^* mice maintained higher core body temperature at 4 °C, and showed higher expression of the thermogenic uncoupling protein 1 (*UCP1*) gene. The ex vivo studies further demonstrated that GHS-R deficient white adipocytes from old mice exhibit increased glucose uptake and lipolysis, promoting lipid mobilization. Despite the fact that the in vivo phenotypes of *aP2*-Cre/*Ghsr^f/f^* mice may not be exclusively determined by GHS-R knockdown in adipose tissues, our data support that GHS-R has cell-autonomous effects in adipocytes. The anabolic effect of GHS-R in adipocytes is more pronounced in aging, which likely contributes to age-associated obesity and insulin resistance.

## 1. Introduction

Aging is often associated with increased obesity and type 2 diabetes. Physical activity is reduced in the elderly and excess energy is stored in adipose tissues, eliciting obesity and insulin resistance [1,2]. The gut peptide ghrelin is the only known circulating orexigenic hormone to stimulate growth hormone (GH) release, enhance appetite, and promote adiposity in humans and rodents [3,4,5,6,7]. We and others have shown that ghrelin’s effects on GH release, appetite and adiposity are mediated through the growth hormone secretagogue receptor, GHS-R [7,8,9]. GHS-R is highly expressed in the pituitary and hypothalamus [9,10,11]; we also found that GHS-R expression in white adipose tissues (WAT) and brown adipose tissues (BAT) is low in young mice but increased in old mice [11,12]. We previously showed that global GHS-R knockout mice (*Ghsr*^−/−^) have reduced body weight and insulin-like growth factor-1 (IGF-1), but normal food intake [7]. We also reported that old *Ghsr*^−/−^ mice are lean and insulin-sensitive, have healthy lipid profiles, and show thermogenic activation in BAT [11,12,13]. Others have shown that GHS-R knockout mice are resistant to diet-induced obesity [14]. These findings together suggest that GHS-R plays an important role in energy metabolism. However, since all these observations were made in global GHS-R knockout mice, it is impossible to assess whether GHS-R has cell-autonomous effects specifically in adipose tissues. Recently, we reported that *synapsin 1*-Cre-mediated neuronal ablation of GHS-R totally prevents high-fat diet-induced obesity [15]; we further reported that specific deletion of GHS-R in Agouti-related protein (AgRP) neurons mitigates diet induced obesity, at least in part, through activation of thermogenesis [16].

Adipocyte Protein 2 (aP2) is a carrier protein for fatty acids primarily expressed in adipocytes. The *aP2*-Cre mice have been widely used to knockout or knockin genes in adipose tissues in mice [17,18,19]. To determine the site(s) of action for GHS-R, we generated *aP2*-Cre-mediated GHS-R knockdown mice (*aP2*-Cre/*Ghsr^f/f^*) by breeding *aP2*-Cre mice with GHS-R^flox/flox^ mice [20]. We investigated the metabolic characteristics, insulin sensitivity and thermogenic activity at different age points of the mouse model. Similar to *Ghsr*^−/−^ mice, the phenotype of young mice was mild; remarkably, though, old *aP2*-Cre/*Ghsr^f/f^* mice showed reduced adiposity, improved insulin sensitivity, and activated thermogenesis. These findings suggest that ghrelin’s effects on adiposity and insulin sensitivity are also attributable to *aP2*-Cre-expressing tissues, and that GHS-R has important metabolic roles in adipose tissues during aging.

## 2. Results

### 2.1. Generation of aP2-Cre/Ghsr^f/f^ Mice

**Figure 1:**
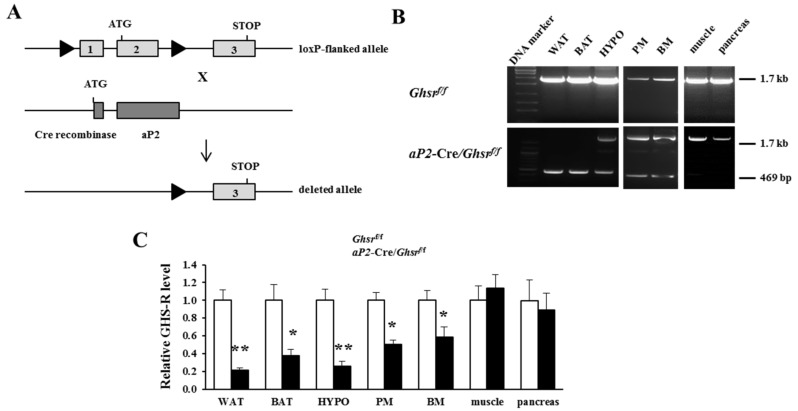
Generation and validation of *aP2*-Cre/*Ghsr^f/f^* mice. (**A**) Schematic diagram of generation of *aP2*-Cre/*Ghsr^f/f^* mice: GHS-R floxed mice were bred with *aP2*-Cre mice to generate *aP2*-Cre/*Ghsr^f/f^* mice; (**B**) polymerase chain reaction (PCR) analysis of genomic DNA isolated from white adipose tissue (WAT), brown adipose tissue (BAT), hypothalamus (HYPO), peritoneal macrophages (PM), bone marrow (BM), skeletal muscle, and pancreas. The 1.7-kb PCR-amplified band indicates the amplification of the GHS-R floxed allele, and the 469 bp PCR-amplified band indicates the deleted GHS-R allele. The 469 bp band was present in WAT, BAT, hypothalamus, PM and BM, but absent in skeletal muscle or pancreas; (**C**) *Ghsr* mRNA expression levels in WAT, BAT, hypothalamus, PM, BM, skeletal muscle and pancreas. *Ghsr* expression was decreased in WAT, BAT, HYPO, PM and BM, but not in other tissues. *n* = 5–9, in each group. * *p* < 0.05, ** *p* < 0.001, *Ghsr^f/f^* vs. *aP2*-Cre/*Ghsr^f/f^*. The results were represented as mean ± standard error of the mean (S.E.M.). The two-tailed Student’s *t*-test was used for statistical analysis.

The generation of the floxed GHS-R allele-containing mice was described earlier [20]. *Ghsr^f/f^* mice were bred with *aP2*-Cre mice [17,18,19] to excise the GHS-R open reading frame in a tissue-specific manner, thus generating *aP2*-Cre*/Ghsr^f/f^* mice (Figure 1A). Two loxP sites were positioned to flank GHS-R exons 1 and 2. Tissue-specific deletion of the *Ghsr* gene was verified by polymerase chain reaction (PCR) analyses of genomic DNA using primers derived from exons 1 and 2: the wild-type allele is 1.7 kilo base pairs (kb), and the deleted allele is 469 base pairs (bp). The 469 bp PCR product was detected in WAT and BAT, but not in skeletal muscle or pancreas (Figure 1B), indicating that exons 1 and 2 of GHS-R were completely deleted in both white and brown adipose tissues, but not in muscle or the pancreas. Both 1.7 kb and 469 bp PCR products were detected in the hypothalamus (HYPO), bone marrow (BM) and peritoneal macrophages (PM), indicative of partial *Ghsr* gene deletion in these tissues/cell types, in agreement with report of aP2 promoter activates neurons and macrophages [17,18]. To further confirm the tissue-specific deletion of GHS-R, *Ghsr* mRNA expression in various tissues was analyzed. *Ghsr* mRNA level in *aP2*-Cre/*Ghsr^f/f^* mice was reduced 80% in WAT and 60% in BAT (Figure 1C and Figure S1). Similarly, q-PCR analysis showed that *Ghsr* mRNA expression in HYPO was also significantly decreased, and modestly decreased expression was detected in PM and BM (Figure 1C and Figure S1).

### 2.2. aP2-Cre Mediated Growth Hormone Secretagogue Receptor (GHS-R) Knockdown Abolishes Ghrelin-Induced GH Secretion and Spontaneous Food Intake

**Figure 2:**
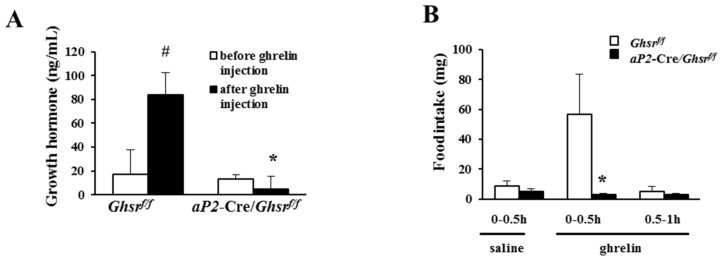
*aP2*-Cre/*Ghsr^f/f^* mice show impaired growth hormone secretion and acute ghrelin-mediated food intake. (**A**) Growth hormone level of the mice before and after *i.p.* injection of 20 μg ghrelin. *n* = 6, # *p* < 0.05, before and after ghrelin injection. * *p* < 0.05, *Ghsr^f/f^* vs. *aP2*-Cre/*Ghsr^f/f^*; (**B**) acute food intake of the mice after *i.p.* injection of either saline or 20 μg ghrelin. *n* = 9–10. * *p* < 0.05, *Ghsr^f/f^* vs. *aP2*-Cre/*Ghsr^f/f^*. The results were represented as mean ± S.E.M. Two-tailed Student’s *t*-test or repeated measures analysis of variance (ANOVA) was used for statistical analysis.

Using global *Ghsr^−/−^* mice, we previously showed that ghrelin-induced GH release and acute food intake is mediated through GHS-R [7]. In the current study, ghrelin was injected intraperitoneally (*i.p.*) into *aP2*-Cre/*Ghsr^f/f^* and *Ghsr^f/f^* mice. GH release after ghrelin injection was significantly increased in *Ghsr^f/f^* mice, but not in *aP2*-Cre/*Ghsr^f/f^* mice (Figure 2A). Similarly, *aP2*-Cre/*Ghsr^f/f^* mice showed significantly reduced food intake after ghrelin injection compared with *Ghsr^f/f^* mice, but the basal levels of GH secretion and food intake were not affected (Figure 2B). This result is in line with reduced GHS-R expression in hypothalamus of *aP2*-Cre/*Ghsr^f/f^* mice (Figure 1C). These data suggest that the acute stimulatory effects of ghrelin on GH release and food intake are mediated by GHS-R in *aP2*-Cre-activated regions, likely involving GHS-R expressing neurons in the hypothalamus.

### 2.3. Old aP2-Cre/Ghsr^f/f^ Mice Have Reduced Adiposity

**Figure 3:**
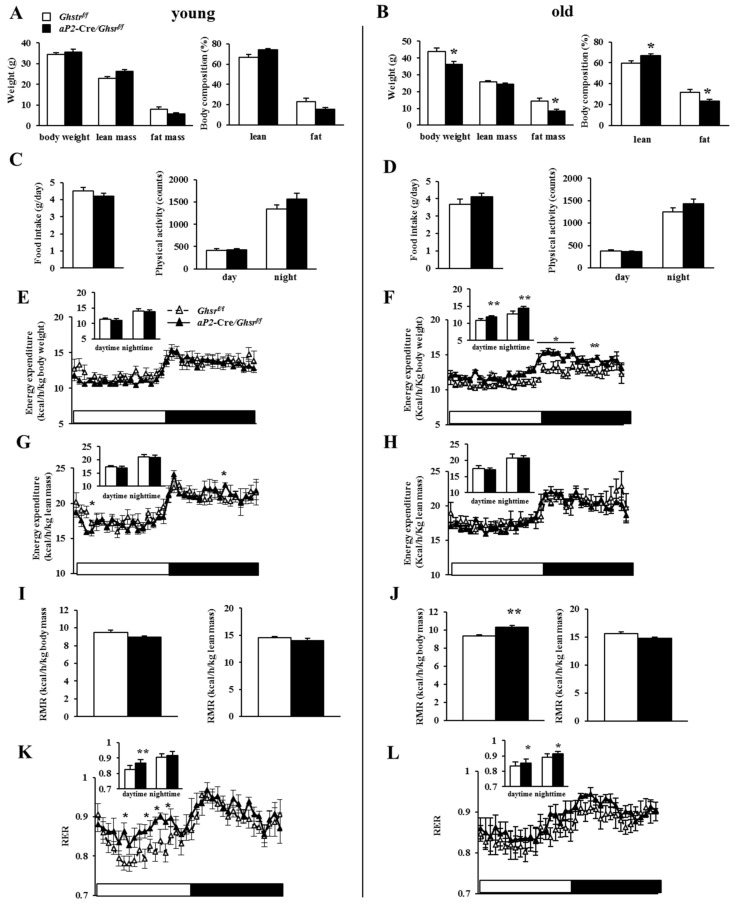
Metabolic profiles of young and old *aP2*-Cre/*Ghsr^f/f^* mice. Indirect calorimetry analysis of young (5–6 months) and old (15–17 months) *Ghsr^f/f^* and *aP2*-Cre/*Ghsr^f/f^* mice. (**A**,**B**) Body weight and body composition of *Ghsr^f/f^* and *aP2*-Cre/*Ghsr^f/f^* mice; (**C**,**D**) daily food intake and physical activity of *Ghsr^f/f^* and *aP2*-Cre/*Ghsr^f/f^* mice. Energy expenditure (EE) by body weight (**E**) or lean mass (**G**), resting metabolic rate (RMR) (**I**), and respiratory exchange ratio (RER) (**K**) of young *Ghsr^f/f^ aP2*-Cre/*Ghsr^f/f^* mice; EE by body weight (**F**) or lean mass (**H**), RMR (**J**) and RER (**L**) of old *Ghsr^f/f^ aP2*-Cre/*Ghsr^f/f^* mice. *n* = 5 in each group. * *p* < 0.05, ** *p* < 0.001 *Ghsr^f/f^* vs. *aP2*-Cre/*Ghsr^f/f^*. The results were represented as mean ± S.E.M. Two-tailed Student’s *t*-test or repeated measures ANOVA was used for statistical analysis.

To further investigate the role of tissue-specific *Ghsr* deletion, we characterized the metabolic phenotype of *aP2*-Cre/*Ghsr^f/f^* mice. In young animals (5–6 months), there was no difference in body weights between *aP2*-Cre/*Ghsr^f/f^* and their age-matched *Ghsr^f/f^* mice (Figure 3A). Sex hormones have important roles in the regulation of adipose proliferation, differentiation and metabolism [21,22,23]. In order to determine whether *aP2*-Cre/*Ghsr^f/f^* mice have sexual dimorphism, we investigated 6-month-old female mice. The data show that body weight and body composition profiles were very similar in males and females (Supplemental Figure S2). To reduce animal usage, we used only male mice for the rest of the study. Our previous studies showed that GHS-R expression in adipose tissues increased with age, and the metabolic phenotype of global *Ghsr* deletion was pronounced only in middle-aged and old mice [11,12]. To determine whether age affects the metabolic phenotype of *aP2*-Cre/*Ghsr^f/f^* mice, we studied the metabolic phenotype of young and old male *aP2*-Cre/*Ghsr^f/f^* mice. Old *aP2*-Cre/*Ghsr^f/f^* mice (15–17 months) showed more pronounced body weight and body fat decreases compared with the age-matched controls (Figure 3B), underscoring the importance of GHS-R expression in tissues where *aP2* gene is expressed during aging.

### 2.4. Old aP2-Cre/Ghsrf/f Mice Have Increased Energy Expenditure and Prefer Carbohydrate as Fuel Source

Our previous study showed that global *Ghsr* deletion in old mice does not affect food intake or physical activity, but increases energy expenditure [11,13]. In the current study, we subjected young (5–6 months) and old (15–17 months) male *aP2*-Cre/*Ghsr^f/f^* and *Ghsr^f/f^* mice to indirect calorimetry analysis to characterize their metabolic profiles. Daily food intake and physical activity were similar between *aP2*-Cre/*Ghsr^f/f^* and *Ghsr^f/f^* mice, regardless of age (Figure 3C,D). The energy expenditure and resting metabolic rate (RMR) of young *aP2*-Cre/*Ghsr^f/f^* mice were similar to control mice, regardless of whether it was normalized to body weight or lean body mass (Figure 3E,G,I). In contrast, the energy expenditure and RMR of old *aP2*-Cre/*Ghsr^f/f^* mice were increased when normalized by body weight, but not by lean mass (Figure 3F,H,J). The data suggest that the lean phenotype of *aP2*-Cre/*Ghsr^f/f^* mice is not likely due to reduced energy intake or elevated physical activity, but increased energy expenditure. Furthermore, the respiratory exchange ratio (RER) was increased in *aP2*-Cre/*Ghsr^f/f^* mice of both age groups, with greater RMR increase during day time resting state in young mice (Figure 3K,L), indicating that the *aP2*-Cre/*Ghsr^f/f^* mice favor carbohydrates as a fuel substrate (as fat reserves in these mice were likely limited due to their leanness). Collectively, the metabolic data show that aP2-specific GHS-R knockdown increases energy expenditure as the mice aged, and the mice grow to in association with preference for carbohydrates as an energy source.

### 2.5. aP2-Cre-Mediated GHS-R Knockdown Mice Have Improved Insulin Sensitivity and Glucose Tolerance

**Figure 4:**
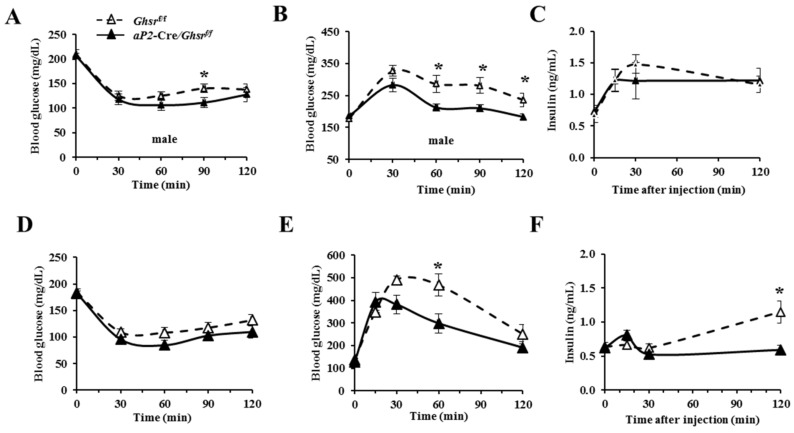
Glycemic profiles of young and old *aP2*-Cre/*Ghsr^f/f^* mice. (**A**) Blood glucose during insulin tolerance tests (ITT), and blood glucose (**B**) and insulin (**C**) during glucose tolerance tests (GTT) of 5–6 months-old young male *Ghsr^f/f^* and *aP2*-Cre/*Ghsr^f/f^* mice; (**D**) blood glucose during ITT, and blood glucose (**E**) and insulin (**F**) during GTT of 15–17 months-old male *Ghsr^f/f^* and *aP2*-Cre/*Ghsr^f/f^* mice. *n* = 7. * *p* < 0.05, *Ghsr^f/f^* vs. *aP2*-Cre/*Ghsr^f/f^*. The results were represented as mean ± S.E.M. Repeated measurement-ANOVA was used for statistical analysis.

Our previous studies revealed that old Ghsr−/− mice have improved insulin sensitivity [11,13], and other studies showed that GHS-R ablation attenuated diet-induced insulin resistance [14,24]. To investigate the role of aP2-specific knockdown of GHS-R in insulin sensitivity, we performed insulin tolerance tests (ITT) and glucose tolerance tests (GTT) on young (5–6 months) and old (15–17 months) male aP2-Cre/Ghsrf/f and Ghsrf/f mice. ITT data showed that aP2-Cre/Ghsrf/f mice were slightly more sensitive to insulin as young mice, but not significant in old mice (Figure 4A,D). During GTT, young aP2-Cre/Ghsrf/f mice showed better glucose clearance compared to Ghsrf/f mice, while the plasma insulin was comparable (Figure 4B,C). In contrast, old aP2-Cre/Ghsrf/f mice showed improved glucose tolerance and reduced plasma insulin (Figure 4E,F). The insulin secretion response during GTT is similar to lower insulin level during GTT we observed in old Ghsr−/− mice [11]. Together, these data suggest that aP2-Cre/Ghsrf/f mice have improved insulin sensitivity and glucose tolerance, similar to that of global GHS-R knockout mice.

### 2.6. aP2-Cre-Mediated GHS-R Knockdown Improves Thermogenesis in Brown Fat, and Enhances Glucose Uptake and Lipolysis in White Fat

**Figure 5:**
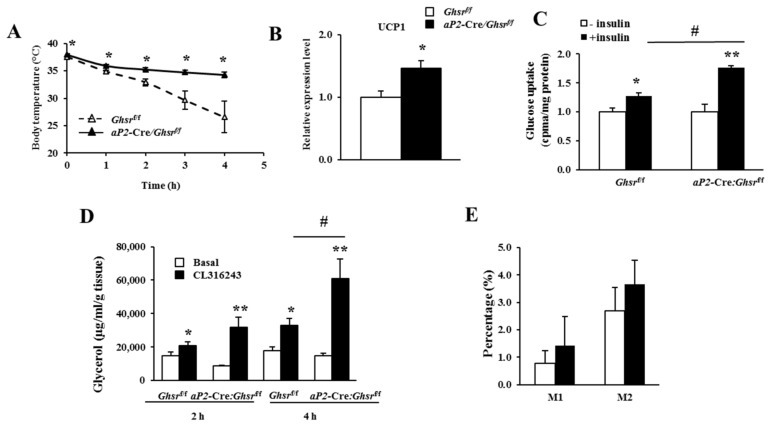
*aP2*-Cre/*Ghsr^f/f^* mice showed improved thermogenesis in brown fat, and increased glucose uptake and lipolysis in white fat. (**A**) Rectal temperature of 10-month-old male *Ghsr^f/f^* and *aP2*-Cre/*Ghsr^f/f^* mice. Mice were individually caged in 4 °C cold room for 4 h, with free access to food and water. The rectal temperatures were collected every hour. *n* = 5–7 in each group; (**B**) *UCP1* mRNA expression levels in the BAT from 16–18 months-old male *Ghsr^f/f^* and *aP2*-Cre/*Ghsr^f/f^* mice. *n* = 9; (**C**) Ex vivo glucose uptake into WAT from 18-months-old male *Ghsr^f/f^* and *aP2*-Cre/*Ghsr^f/f^* mice. *n* = 5. # *p* < 0.05, basal vs. insulin stimulation; (**D**) ex vivo lipolysis in WAT from 18-months-old male *Ghsr^f/f^* and *aP2*-Cre/*Ghsr^f/f^* mice. *n* = 5; (**E**) flow cytometry analysis of peritoneal macrophages of 18-months-old *Ghsr^f/f^* and *aP2*-Cre/*Ghsr^f/f^* mice. *n* = 4–5. # *p* < 0.05, basal vs. CL316,243 treatment. * *p* < 0.05, ** *p* < 0.001, *Ghsr^f/f^* vs. *aP2*-Cre/*Ghsr^f/f^*. The results were represented as mean ± S.E.M. Two-tailed Student’s *t*-test or repeated measures ANOVA was used for statistical analysis.

One of the key characteristics we observed in old *Ghsr*^−/−^ mice was increased thermogenesis in BAT. To compare cold-induced thermogenesis, we measured the rectal temperatures of *aP2*-Cre/*Ghsr^f/f^* and control *Ghsr^f/f^* mice at 4 °C cold exposure. Rectal temperature of *aP2*-Cre/*Ghsr^f/f^* mice at basal level was higher than that of *Ghsr^f/f^* mice (38.00 °C vs. 37.48 °C); and the difference became more pronounced at an ambient temperature of 4 °C (Figure 5A). The results suggest that *aP2*-Cre/*Ghsr^f/f^* mice have increased thermogenesis, especially during cold exposure. BAT is a major organ for non-shivering thermogenesis, and UCP1 is the key regulator of thermogenesis, so we then compared UCP1 expression in BAT. Indeed, *UCP1* gene expression in BAT of *aP2*-Cre/*Ghsr^f/f^* mice was significantly increased (Figure 5B), indicating that reduced thermogenesis is a major metabolic consequence of GHS-R activation in BAT.

WAT is the major organ to uptake glucose and store energy. We compared glucose uptake capacity using mature white adipocytes. Consistent with GTT, adipocytes from epididymal fat of *aP2*-Cre/*Ghsr^f/f^* mice had significantly-increased glucose uptake under insulin-stimulated condition, and were more sensitive to insulin (Figure 5C). Lipolysis is a key mechanism for releasing free fatty acid from WAT as an energy source [25]. We next examined ex vivo lipolysis capacity of epididymal WAT from *Ghsr^f/f^* and *aP2*-Cre/*Ghsr^f/f^* mice. The results showed *aP2*-Cre-mediated GHS-R knockdown did not change lipolysis in WAT at basal condition, but promoted lipolysis under treatment of β3-adrenergic receptor agonist CL316,243 (Figure 5D). The ex vivo adipocyte studies indicate that GHS-R has direct effects in adipocytes, and GHS-R deficient adipocytes reveal increased glucose uptake and lipolysis under stimulated conditions.

Since *aP2*-Cre/*Ghsr^f/f^* mice show partial deletion in macrophages, we studied peritoneal macrophages of old *Ghsr^f/f^* and *aP2*-Cre/*Ghsr^f/f^* mice using flow cytometry. The results showed no alteration of M1-like macrophages (F4/80^+^CD11c^+^CD206^−^) nor of M2-like macrophages (F4/80^+^CD11c^−^CD206^+^) in *aP2*-Cre/*Ghsr^f/f^* mice (Figure 5E). Thus, even though there is partial knockdown of GHS-R in macrophages, it does not appear to affect the macrophage phenotype. Thus, we believe that partial gene knockdown of GHS-R in macrophages may not make a major contribution to affect the metabolic phenotype of *aP2*-Cre/*Ghsr^f/f^* mice. Taken together, GHS-R knockdown increases thermogenesis in BAT, and enhances glucose uptake and lipolysis in WAT, in line with the lean phenotype.

## 3. Discussion

Our previous studies suggest that GHS-R is an important thermogenic regulator during aging, and that GHS-R has direct effects in brown adipocytes [11,12]. In the present study, we generated *aP2*-Cre/*Ghsr^f/f^* mice and aimed to study the function of GHS-R in adipose tissues as they age. Identification of the aP2 promoter as a driver for gene expression in adipocytes was a major step forward for adipose tissue research [26]. *aP2* gene promoter-driven Cre mice have long been used in adipose tissue-specific transgenic mouse studies for both gene knockout and knockin [17,27,28]. The *aP2* gene is found predominantly in mature adipocytes, which makes it a good candidate to drive high-level transgene expression in adipocytes. However, it has been recently found that the *aP2*-Cre activity can also be detected in cell types such as macrophages [29] and the brain [18,19,30]. Our validation data showed that *aP2*-Cre/*Ghsr^f/f^* mice do have ectopic GHS-R knockdown in the hypothalamus and macrophages. Partial GHS-R genomic DNA deletion was detected in hypothalamus, peritoneal macrophages and bone marrow and reduced mRNA expression of GHS-R was detected in these tissues, as well as in WAT and BAT. Thus, while *aP2*-Cre primarily targets adipose tissues, but due to the ectopic activation of *aP2*-Cre, it is possible that the phenotypes of *aP2*-Cre/*Ghsr^f/f^* mice we detected are also attributable to partial GHS-R deletion in the brain and/or macrophages. Even though *aP2*-Cre/*Ghsr^f/f^* mice are not exclusively adipose tissue-specific GHS-R inactivation per se, the current study allows us to assess the effects of GHS-R in adipocytes, macrophages, and/or brain, providing more specific tissue-specific assessment than the early globally-ablated mice.

The *aP2*-Cre/*Ghsr^f/f^* mice showed partial knockdown of GHS-R expression in the hypothalamus, which may result in reduced receptor function that dampens ghrelin’s effects on ghrelin-induced GH release and ghrelin-induced food intake. The *aP2*-Cre-mediated knockdown of GHS-R abolishes ghrelin-induced GH secretion and acute food intake, indicating that GHS-R reduction in the hypothalamus of the mice does indeed affect ghrelin’s central functions. However, we were not able to detect a change in total energy intake. This is in line with our observations in *Ghsr^−/−^* mice, further supporting the notion that GHS-R is not essential for long-term energy intake. We observed that the body fat mass of *aP2*-Cre/*Ghsr^f/f^* mice was significantly reduced, without any change in total food consumption or physical activity, indicating that the metabolic phenotype of *aP2*-Cre/*Ghsr^f/f^* mice is likely primarily due to increased energy expenditure. Consistently, the energy expenditure of old *aP2*-Cre/*Ghsr^f/f^* mice was increased. Old *aP2*-Cre/*Ghsr^f/f^* mice were able to maintain higher body temperature under cold exposure, and *UCP1* expression was increased in BAT. This suggests that the lean and insulin-sensitive phenotype observed in *aP2*-Cre/*Ghsr^f/f^* mice is, at least in part, due to enhanced thermogenesis induced by GHS-R deficiency in adipose tissues and/or the brain. Since partial GHS-R knockdown in peritoneal macrophages and bone marrow did not affect the macrophage polarization phenotype, we speculate that the partial gene knockdown of GHS-R in macrophages may not have major impact on the overall metabolic phenotype of *aP2*-Cre/*Ghsr^f/f^* mice.

Previous studies showed that administration of either ghrelin or a synthetic ghrelin agonist increases RER in rats and mice [4,31]. Conversely, we showed that old *Ghsr^−/−^* mice have increased RER [11,13]. These two conflicting reports suggest that the effects of ghrelin signaling on fuel substrate preferences are more complex. Consistent with our previous data in *Ghsr^−/−^* mice, we detected higher RER in *aP2*-Cre/*Ghsr^f/f^* mice relative to the controls, especially during the light phase of the cycle. The higher RER in *aP2*-Cre/*Ghsr^f/f^* mice indicates that the mice favor carbohydrate as a fuel substrate; the reduced fat reserves in the lean *aP2*-Cre/*Ghsr^f/f^* mice may obligate them to utilize carbohydrates instead of fat. There is a difference in insulin levels during GTT between global *Ghsr^−/−^* and *aP2*-Cre/*Ghsr^f/f^* mice; this may be due to the knockdown of GHS-R in the pancreas in *Ghsr^−/−^* but not in *aP2*-Cre/*Ghsr^f/f^* mice.

Since *aP2*-Cre/*Ghsr^f/f^* mice largely emulate the metabolic phenotype of *Ghsr^−/−^* mice, suggesting that GHS-R in adipose tissues, macrophages, and/or brain is important for its metabolic regulation. While we cannot conclude that in vivo phenotype of *aP2*-Cre/*Ghsr^f/f^* mice is determined by GHS-R knockdown in adipose tissues alone, it important to note that our ex vivo studies clearly indicate that GHS-R has cell-autonomous effects in adipocytes, regulating both lipid and glycose metabolism. Although several studies show that ghrelin and GHS-R have direct effects in adipocytes, it is still a controversial topic [32,33,34,35]. A*diponectin*-Cre has been considered as a better driver to specifically target adipocytes [36], hence it would be informative to study *adiponectin*-Cre-mediated GHS-R knockout to further confirm the adipose-specific roles of GHS-R. 

Collectively, our current data showed that old *aP2*-Cre/*Ghsr^f/f^* mice have improved whole-body insulin sensitivity and glucose tolerance, which further supports our previous observations in old global *Ghsr^−/−^* mice that ghrelin signaling pathway plays an important role in glucose homeostasis. Our previous studies showed that expression of GHS-R in adipose tissue is increased during aging, and the lean and insulin-sensitive phenotype of *Ghsr^−/−^* mice becomes more prominent when animals are old [11]. Similarly, old (not young) *aP2*-Cre/*Ghsr^f/f^* mice have a more pronounced metabolic phenotype. Our data collectively support the notion that GHS-R has cell-autonomous effects in adipose tissues, and its adipose effects are augmented in aging.

## 4. Materials and Methods

### 4.1. Animals

*aP2*-Cre mice (Stock number 005069, B6.Cg-Tg (Fabp4-cre)1Rev/J) were obtained from Jackson Laboratory. Generation of floxed GHS-R allele-containing mice has been described previously [20]. We removed the FRT-PGK-neo-FRT cassette by breeding them with FLP mice, and then backcrossed them for 10 generations onto C57BL/6J background. Mice were bred and housed in a pathogen-free facility at Baylor College of Medicine. Animals were housed under controlled temperature (23 ± 1 °C) and 12 h light-dark cycle with free access to food and water. Normal chow diet (2920X, 16% of calories from fat, 60% from carbohydrates, 24% from protein) was purchased from Harlan-Teklad (Madison, WI, USA). All experiments were approved by the Animal Care and Research Committee of the Baylor College of Medicine. Age-matched male and female wild-type (WT), *aP2*-Cre, *Ghsr^f/f^*, and *aP2*-Cre/*Ghsr^f/f^* mice were used in the studies, including young (5–6 months) and old (15–17 months) cohorts. All animal experiments have been approved by Institutional Animal Care and Use Committee at Texas A&M University with the approval code as AUP IACUC 2016-0292.

### 4.2. Genotyping of aP2-Cre/Ghsr^f/f^ Mice

Tail DNA was extracted using Direct PCR Lysis Reagent (Tail) (Viagen Biotech, Los Angeles, CA, USA), following the manufacturer’s instructions. For *Ghsr^f/f^* genotyping, forward (5′-CTGAAGGCATCTTTCACTACG-3′) and reverse (5′-TGGGGGTGCGAACATTAGC-3′) primers were used. For *aP2*-Cre genotyping, the forward (5′-CTAAGTCCAGTGATCATTGCCAGGGA-3′) and the reverse (5′-CCGGCAAACGGACAGAAGCA-3′) primers were used.

### 4.3. Tissue DNA Extraction and Cre Excision Analysis

DNA from different tissues was extracted as described [37]. For Cre excision, forward (5′-CTGAAGGCATCTTTCACTACG-3′) and reverse (5′-ACATATTCTATGTGAGGCACC-3′) primers were used for PCR amplification. The PCR products were then electrophoresed on 2.5% agarose gel.

### 4.4. Real-Time Polymerase Chain Reaction (RT-PCR)

Total RNA of tissues was isolated using TRIzol Reagent (Invitrogen, Carlsbad, CA, USA) following the manufacturer’s instructions. RNA was treated with DNase and run on the gels to validate the purity and quality. The cDNA was synthesized from 1 μg RNA using the SuperScript III First-Strand Synthesis System for real-time PCR (RT-PCR) (Invitrogen). Real-time PCR was performed on a Bio-Rad qPCR machine using the SYBR Green PCR Master Mix, according to the manufacturer’s recommended procedures. The primers were as follows: *GHS-R* forward primer 5′-GGACCAGAACCACAAACAGACA-3′, *GHS-R* reverse primer 5′-CAGCAGAGGATGAAAGCAAACA-3′; This primer set flanks the intron, which allows us to distinguish its expression from GHS-R 1b. *UCP1* forward primer 5′-GTGAAGGTCAGAATGCAAGC-3′, *UCP1* reverse primer 5′-AGGGCCCCCTTCATGAGGTC-3′. *18S* and *β*-actin were used as housekeeping genes.

### 4.5. Body Composition and Indirect Calorimetry

Body composition (fat and lean mass) of mice was measured by an Echo MRI-100 whole-body composition analyzer (Echo Medical Systems, Houston, TX, USA), following the manufacturer’s instructions as previously described [11,13]. Metabolic parameters were obtained by using a Comprehensive Laboratory Animal Monitoring System (CLAMS, Columbus Instruments, Columbus, OH, USA) for 6 days. The mice were individually caged in chambers and given free access to regular diet and water for 1-week prior to CLAMS tests. The first 2 days of CLAMS was considered the acclimation phase, and data for the next 3 days were analyzed. Oxygen consumption (VO_2_) (mL/h), carbon dioxide production (VCO_2_) (mL/h), and locomotor activity (infrared beam-break counts) were recorded. RER and energy expenditure (EE, or heat generation) were calculated from VO_2_ and VCO_2_ gas exchange data as follows: RER = VCO_2_/VO_2_ and EE = (3.815 + 1.232 × RER) × VO_2,_ respectively. Energy expenditure was then normalized to either body weight or lean body mass. Locomotor activity was measured on x- and z-axes by the counts of beam-breaks during the recording period. The mice were fasted from 6 am to 2 pm on the last day, and the three lowest energy expenditure readings between 10 am to 2 pm were used to assess RMR.

### 4.6. Insulin Tolerance Test (ITT) and Glucose Tolerance Test (GTT)

ITTs and GTTs were carried out using young (5–6 months) and old (15–17 months) male mice. After 6 h fast, blood glucose was measured using a OneTouch Ultra blood glucose meter with LifeScan test strips. Mice then received an *i.p.* injection of human insulin (Eli Lilly, Indianapolis, IN, USA) at a dose of 1.0 U/kg of body weight. Tail blood glucose concentration was measured at 0, 30, 60, 90 and 120 min after injections. The GTT was carried out after overnight fast, the mice received an *i.p*. injection of glucose solution (Sigma-Aldrich, St. Louis, MO, USA) at a dose of 2.0 g/kg body weight. The blood glucose was measured at 0, 15, 30, 60 and 120 min after injections, and blood samples were collected for insulin analysis at 0, 15, 30 and 120 min after injections.

### 4.7. Rectal Temperature

Rectal temperatures were measured as described [38]. Briefly, basal body temperature was collected, using a TH-8 temperature Monitor System (Physitemp, Clifton, NJ, USA). The mice were kept in a 4 °C cold room with free access to food and water; the rectal temperatures were monitored hourly, and the mice experiencing hypothermia were returned immediately to normal housing temperature for recovery.

### 4.8. Glucose Uptake

The glucose uptake was measured ex vivo in adipocytes isolated from epididymal WAT as previously described [39,40]. Briefly, dissected adipose tissue was minced into tiny pieces and digested with 1 mg/mL collagenase type1 (Worthington Chemicals, Lakewood, NJ, USA) in serum-free media containing 20 mg/mL BSA. After incubation at 37 °C for 45 min, the cells were filtered and washed twice. Afterward, the adipocyte pellets were re-suspended and ready to be tested. The isolated adipocytes were first cultured in Dulbecco’s Modified Eagle Medium (DMEM) supplied with 0.5% fatty acid-free BSA (Sigma-Aldrich). For glucose uptake, the medium was replaced with Krebs Ringer Hepes (KRH) buffer (1.2 M NaCl, 50 mM KCl, 10 mM KH_2_PO_4_, 6 mM MgSO_4_, 10 mM CaCl_2_) containing insulin (100 nM), and incubated for 20 min at 37 °C. Then, 50 μL of 10 mM 2-deoxyl glucose and 1 μCi/μL 2-deoxy-D-[^3^H]-glucose was added and further incubated for 10 min. The cells were then washed twice in ice-cold KRH and lysed in 1 mL of 0.1% sodium dodecyl sulfate. Radioactivity was determined in 5 mL of scintillant using a scintillation counter. Glucose uptake was represented by radioactivity normalized by protein concentration from a BCA (bicinchoninic acid) assay (Thermo Scientific, Waltham, MA, USA).

### 4.9. Ex Vivo Lipolysis

The lipolysis activity of WAT was measured using ex vivo lipolysis assay as described [12]. Briefly, epididymal WAT was dissected and chopped into tiny pieces with scissors in DMEM with 0.5% fatty acid-free BSA. The tissues were incubated at 37 °C with 10 μM CL316,243 (Sigma-Aldrich) as stimulated condition or dimethyl sulfoxide (DMSO) as basal condition. Culture medium was collected at 2 h and 4 h after the incubation. The medium was heated at 85 °C for 10 min. After centrifugation at 4000 g for 5 min at room temperature, clear supernatant was transferred to a new tube, and 10 μL medium was used to measure free glycerol content using Free Glycerol Reagent (Sigma-Aldrich). Lipolysis activity was calculated as glycerol concentrations normalized by weight of the tissue.

### 4.10. Ghrelin-induced GH Secretion and Food Intake

For ghrelin-induced GH measurement: mice were *i.p.* injected with 50 mg/kg pentobarbital. 15 min later, 20 μg ghrelin in 100 μL of physiologic saline were *i.p.* injected. Blood samples were collected from the tail at 0, and 5 min after ghrelin administration. The plasma concentration of GH was determined with a GH Rat/Mouse Hormone RIA kit (Millipore Corporation, Billerica, MA, USA). For ghrelin-induced acute food intake: mice were fasted for 3 h in the morning (6 am–9 am), then *i.p.* injected with 100 μL physiologic saline and food intake was measured at 0.5 h after the injection. The same mice were then *i.p*. injected with 100 μL physiologic saline containing 20 μg ghrelin. Food intake was measured at 0.5 and 1 h after the ghrelin injection, respectively.

### 4.11. Flow Cytometry Analysis

PM were isolated as described previously [41]. Briefly, about 5 mL of cold phosphate buffer saline (PBS) was injected into mouse abdominal cavities. After vigorously shaking the mice for 2 min, solution in abdominal cavity (the PBS containing PM) was carefully collected. PM was obtained by centrifugation at 1000 *g* for 5 min. For flow cytometry analysis, equal amounts of the PM cells (1 × 10^6^ in 100 μL PBS) were incubated with appropriate antibodies; PE anti-mouse F4/80 antigen (eBioscience, San Diego, CA, USA), FITC anti-mouse CD11c antigen (BD Bioscience, San Jose, CA, USA), and APC anti-mouse CD206 antigen (BD Bioscience). The flow cytometry data were collected using a FACScan and analyzed with Cell Quest software (BD Biosciences). Macrophages labeled with F4/80^+^CD11c^+^CD206^−^ were counted as pro-inflammatory M1-like macrophages and those labeled with F4/80^+^CD11_C_^−^CD206^+^ were counted as anti-inflammatory M2-like macrophages.

### 4.12. Statistical Analysis

Repeated measures analysis of variance (ANOVA) and/or the two-tailed Student’s *t*-test were recruited to determine statistical significance between genotypes or treatments. Sidak’s multiple comparisons test was used for post-hoc analysis. The results were represented as: mean ± S.E.M. Statistical significance was set to a minimum of *p* < 0.05.

## 5. Conclusions

In conclusion, we generated a novel mouse model with *aP2*-Cre-mediated GHS-R inactivation in adipose tissues, with partial GHS-R knockdown in macrophages and the brain. Old *aP2*-Cre/*Ghsr^f/f^* mice exhibited a lean and insulin-sensitive phenotype, showing increased thermogenesis leading to increased energy expenditure, but with no change in total food intake or physical activity. These findings suggest that GHS-R in *aP2*-activating tissues plays an important role in the regulation of adiposity and insulin sensitivity in aging. Moreover, our data suggest that GHS-R has direct effects in adipocytes, and is involved in thermogenesis in brown fat as well as glucose uptake and lipolysis in white fat. Our results indicate GHS-R expression in adipose tissues is likely involved in the pathogenesis of obesity and insulin resistance in aging, GHS-R inactivation in adipose tissues may provide a novel therapeutic strategy for the control of aging-related obesity.

## Supplementary Materials

The following are available online at http://www.mdpi.com/1422-0067/19/10/3002/s1.

## Abbreviations

AgRP	Agouti-related protein
aP2	Adipocyte Protein 2
BAT	Brown adipose tissue
BM	Bone marrow
EE	Energy expenditure
GH	Growth hormone
GHS-R	Growth hormone secretagogue receptor
GTT	Glucose tolerance test
HYPO	Hypothalamus
IGF-1	Insulin-like growth factor-1
ITT	Insulin tolerance test
PM	Peritoneal macrophages
RER	Respiratory exchange ratio
RMR	Resting metabolic rate
UCP-1	Uncoupling protein-1
WAT	White adipose tissue
