# Supplementary material for: aP2-Cre Mediated Ablation of GHS-R Attenuates Adiposity and Improves Insulin Sensitivity during Aging
Source: Int J Mol Sci. 2018 Oct 1;19(10):3002. doi: 10.3390/ijms19103002 (PMC6213105; doi:10.3390/ijms19103002)
Supplement: Supplementary file 1 [file ijms-19-03002-s001.pdf]

International Journal of *Molecular Sciences*

MDPI

Supplementary Material

aP2-Cre Mediated Ablation of GHS-R Attenuates Adiposity and Improves Insulin Sensitivity during Aging

Ligen Lin [1,2], Jong Han Lee [1,3], Ruitao Wang [4], Ru Wang [5], David Sheikh-Hamad [6], Qun S. Zang [7] and Yuxiang Sun [1,8,*]

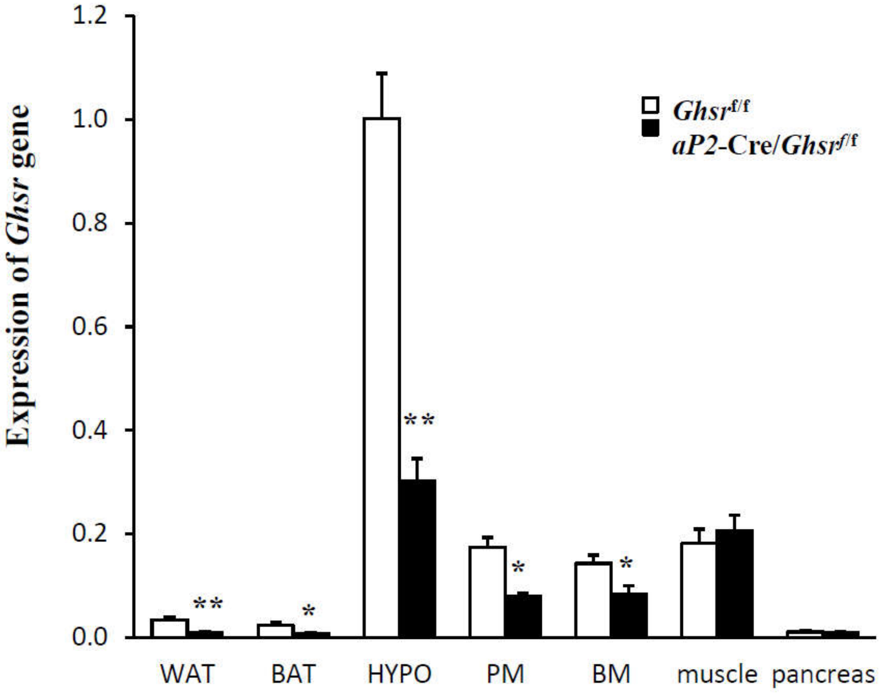

Supplemental Figure S1. Expression of Ghsr gene in different tissues from *Ghsr$^{f/f}$* and *aP2-Cre/Ghsr$^{f/f}$* mice. WAT: white adipose tissue; BAT: brown adipose tissue; HYPO: hypothalamus; PM: peritoneal macrophages; BM: bone marrow. n = 6–12 in each group. * $P < 0.05$, ** $P < 0.001$, *Ghsr$^{f/f}$ vs. aP2-Cre/Ghsr$^{f/f}$*.

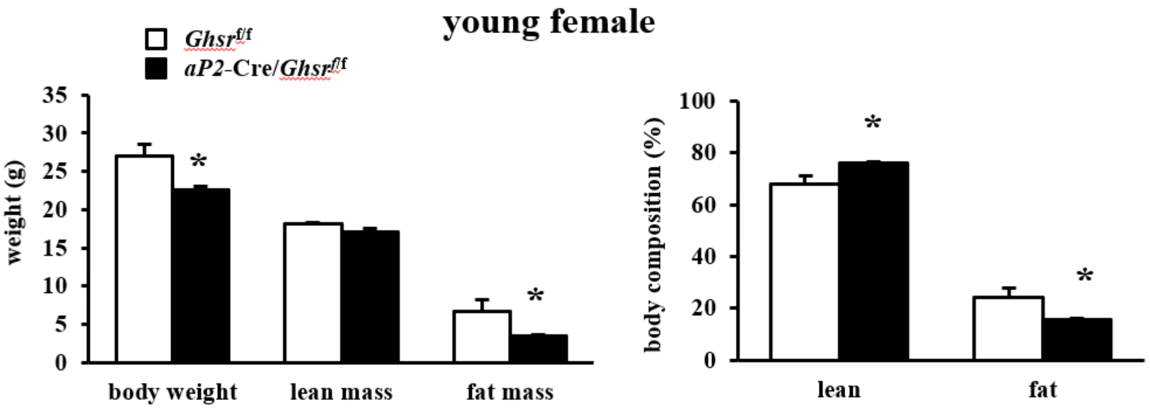

Supplemental Figure S2. Metabolic profile of female *aP2*-Cre/*Ghsr$^{f/f}$* mice. Body weight and body composition of 5–6 month-old female *Ghsr$^{f/f}$* and *aP2*-Cre/*Ghsr$^{f/f}$* mice. n = 5 in each group. * $P < 0.05$, Ghsr$^{f/f}$ *vs. aP2*-Cre/*Ghsr$^{f/f}$*.
